# A nonlinear bi-level programming approach for product portfolio management

**DOI:** 10.1186/s40064-016-2421-0

**Published:** 2016-06-16

**Authors:** Shuang Ma

**Affiliations:** School of Management and Economics, Beijing Institute of Technology, Beijing, 100081 China

**Keywords:** Product portfolio management, Nonlinear bi-level programming, Leader–follower joint optimization, Bi-level nested genetic algorithm

## Abstract

Product portfolio management (PPM) is a critical decision-making for companies across various industries in today’s competitive environment. Traditional studies on PPM problem have been motivated toward engineering feasibilities and marketing which relatively pay less attention to other competitors’ actions and the competitive relations, especially in mathematical optimization domain. The key challenge lies in that how to construct a mathematical optimization model to describe this Stackelberg game-based leader–follower PPM problem and the competitive relations between them. The primary work of this paper is the representation of a decision framework and the optimization model to leverage the PPM problem of leader and follower. A nonlinear, integer bi-level programming model is developed based on the decision framework. Furthermore, a bi-level nested genetic algorithm is put forward to solve this nonlinear bi-level programming model for leader–follower PPM problem. A case study of notebook computer product portfolio optimization is reported. Results and analyses reveal that the leader–follower bi-level optimization model is robust and can empower product portfolio optimization.

## Background

In the highly competitive environment, an important strategic decision for an organization is to optimize its product portfolio to stimulate sales and increase revenues (Ho and Tang 1998). Therefore, optimal product portfolios are interesting for many people over last decades. PPM is a dynamic decision process that analyzes production ability and market potential, and hence determines the optimal portfolio of products with consideration of profit maximization (McNally et al. 2009). PPM is used to develop a set of products with their diversity such as attribute, attribute level, also simultaneously considering product performance, competitive environment information, engineering requirement, manufacturing procedure, and etc. (Cooper et al. 1999). Thus, PPM problem is not a single-objective optimization but a multi-objective combinatorial optimization problem.

In recent years, with the development of product portfolio design, researches in different perspectives of product portfolio optimization have been raised. Such as, considering product portfolio planning with the view of customer–engineering interaction (Jiao and Zhang 2005); using a data mining method to manage a portfolio of products in the cross-domain (Pachidi et al. 2014), but with little concerns in competitive environment or competitive relationship in the product portfolio optimization research.

According to Stackelberg, each firm in a competitive market can be a leader or a follower. The leader is a decision-maker and the follower is characterized as a firm that behaves according to a Nash reaction function (Colson et al. 2007). Therefore, the leader’s PPM optimization results could be very different from the follower’s because of their disparate decision-making positions. Optimization for PPM problem with competitive environment considerations is not an optional issue, but a targeted one. In this paper, a hierarchical joint optimization model is developed in line with bi-level programming, in which focal manufacturer plays as a leader while others act as followers.

The rest of the paper proceeds as follows. Next section reviews related work regarding PPM and bi-level programming. PPM with competitive considerations is formulated as a leader–follower joint optimization problem in “Problem description” section. “Bi-level joint optimization” section elaborates the formulation of a bi-level optimization model for PPM. “Model solution” section develops a bi-level nested genetic algorithm for efficient solution of the hierarchical joint optimization model. A case study of notebook computer is reported in “Case study” section and paper concludes in “Conclusions” section.

## Related work

### PPM

PPM problem is defined as a dynamic decision-making process in which some optimization criteria should be finished, such as share-of-choice (Sadeghi and Zandieh 2011). Four main goals of PPM are summarized by Cooper et al. (2002): (1) Value maximization, (2) Strategic choice, (3) New product and technology choices and (4) Balancing resources. First, value maximization is to optimize resource allocation to gain the maximal value with this product portfolio. Strategic choice aims to ensure a right decision on the business strategy. And new product and technology choices link to what kind of active products, R&D projects and new technology are updated and revised. Balancing resources asks to ensure right number of projects (products) with limited resources and allocate resources between projects or products.

A sizeable body of research on PPM has been reported over the last decade, generally can be divided into four categories with different optimization goals (Otten et al. 2015). With value maximization goal, the fiscal optimization should be performed, and methods are much various, for example, Expected Commercial Value (ECV) method, Productivity Index (PI) and Return on Investment (ROI) method (Cooper et al. 2001; Dickinson et al. 2001). As for the second goal, Bai and Sarkis (2013) pointed out that strategic alignment is the most important success factor for developing business processes and performance management. And a method for achieving strategic alignment with its product portfolio is called the strategic bucket method which is defined as a financial support for a new product development in line with an extraordinary strategy (Chao and Kavadias 2007). The third perspective is new product and technology choices. According to Barczak et al. (2009), performance of a company is coupled with the rate of innovative new product and technology practice. Meanwhile, current literature on PPM covers new product and technology choices with lifecycle consideration, and it’s just like a project in new product design process (Tolonen et al. 2015). Balancing resources can be clarified by terms and parameters such as time, risk, markets and technologies (Tolonen et al. 2015). This process can be accomplished by developing a resource capacity analysis with the quantification of product’s demand for resources versus the availability of them (Otten et al. 2015).

### Bi-level programming

The theoretic ground form of bi-level programming formulation originates from the Stackelberg games (von Stackelberg 1952). Bi-level optimization refers to a mathematical programming which contains a sub-optimization problem as its constraint, first studied and proposed by Bracken and McGill (1973). Bi-level programming theory rapidly becomes an important branch in mathematical programming field because of its abstract of an essential class of hierarchical decision-making problems including Stackelberg Game. Although as a generalized form of mathematical programming, bi-level programming is very different from ordinary mathematical programming. Bi-level optimization problems are complex and often belong to a higher complexity class than their corresponding single-level relaxations (Sakawa et al. 2011). For example, Jeroslow shows that even a linear bi-level programming problem is NP-hard (Jeroslow 1985). Du and Wang (2009) point out that when upper-level constraints contain the optimization solution of the lower-level problem the feasible region may become discontinuous. Bi-level programming is also much more complicated due to difficulties in solving the problems (Christiansen et al. 2001). Traditional solution approaches include vertex enumeration algorithms and methods based on Kuhn–Tucker (KKT) conditions or penalty functions.

Recent trends of bi-level programming mainly revolve around computing and solving this critical problem. One focus of researches is on the theoretical solutions. For example, Ye and Zhu (2010) investigate for bi-level programming solutions based on the value functions. While another one focuses on the approximation solutions, for instance, Sakawa et al. (2011) work on fuzzy interaction-based satisfaction solutions for bi-level programming. The leader–follower Stackelberg and bi-level optimization have been applied in a number of fields, including networks (van Hoesel 2008), advertising (Aust and Buscher 2012), retailer supply chains (Xiao et al. 2014; Esmaeili et al. 2009), and supply chain contracts (Chen et al. 2012).

## Problem description

Consistent with a Stackelberg game, product portfolio management can be formulated as a hierarchical optimization problem consisting of one upper-level and one or more lower-level decision-making agents. The former plays a leader’s role, denoted as *F*, whilst the latter acts as a follower, denoted as *f*. The leader’s decision-making results will serve as the constraints of followers’ optimization problem, whereas the followers feed their optimal solutions back to the leader for adjustment of the leader’s decision. For the leader–follower joint optimization problem, the leader and followers can be the real designers or design teams, or any virtual decision makers.

**Fig. 1:**
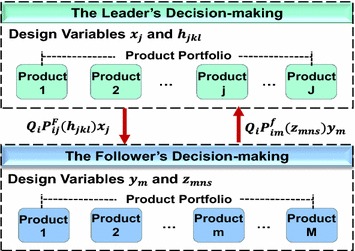
Bi-level decision-making between leader and follower

Figure 1 illustrates the bi-level decision model for product portfolio optimization. The leader *F* determines its selections firstly, and the follower *f* adjusts its product portfolio subjecting to the feasible regions given by the leader.

Assuming that there is one leader and only one follower in the market place to simplify this bi-level optimization problem. The total number of products in leader’s product family is *J* and the follower’s is *M*, which are separately defined as a set {*R*_*j*_} (j = 1, 2, …, J) and {*r*_*m*_} (m = 1, 2, …, M). With variant attributes and attribute levels, utility and cost of each product is very different. The optimal solutions will be sent to the follower as vectors *X* and *H* after the leader’s decision-making according to marketing and engineering considerations, then the follower optimizes its PPM problem and sends solutions to the leader as a feedback. I construct a criterion of market share constraint as the coordinated mechanism for this bi-level optimization problem to develop a more satisfactory product portfolio scheme for both leader and follower.

## Bi-level joint optimization

As described in Chapter 3, optimization for PPM problem aims to provide an optimal set of products with mixture of attributes into the target marketplace for both leader and follower (Jiao and Zhang 2005). In the following, I address how to formulate the bi-level programming model containing objective functions and constraints for this optimization.

### Upper-level PPM optimization

The upper-level deals with F-PPM problem by selecting appropriate combination from a number of *J* products. The decision variable of upper-level is *x*_*j*_ (j = 1, 2, …, J) and {*h*_*jkl*_ (k = 1, 2, …, J; l = 1, 2, …, L), both are binary variables. Such that *x*_*j*_ = 1 indicates that product *R*_*j*_ is selected, and *x*_*j*_ = 0 not. And *h*_*jkl*_ = 1 means that the *l*th attribute level of the *k*th attribute is contained in the *j*th product and *h*_*jkl*_ = 0 not. According to Jiao and Zhang (2005), economics surplus should be leveraged from both marketing and engineering. This paper proposes to use an objective of shared surplus to optimize the PPM problem, as shown in Eq. (1).

1 $$\mathop {\hbox{max} }\limits_{{x_{j} ,h_{jkl} }} F = \mathop \sum \limits_{i = 1}^{I} \mathop \sum \limits_{j = 1}^{J} \frac{{U_{ij}^{F} }}{{C_{j}^{F} }}P_{ij}^{F} Q_{i} x_{j}$$

Assume $U_{ij}^{F}$ is the utility of the *i*th segment for the *j*th product, which is a linear function of the part-worth utilities of the attribute levels of product *R*_*j*_, as shown in Eq. (2).

2 $$U_{ij}^{F} = \mathop \sum \limits_{k = 1}^{K} \mathop \sum \limits_{l = 1}^{{L_{k} }} w_{jk}^{F} u_{ikl}^{F} h_{jkl} + \pi_{ij}^{F} + \varepsilon_{ij}^{F} , \quad \forall i,j$$

where $u_{ikl}^{F}$ is the customer perceived utility of the *i*th segment for the *l*th level of the *k*th attribute; $w_{jk}^{F}$ is the weight of the *k*th attribute; $\pi_{ij}^{F}$ is a constant related to the composite utility of product variant *R*_*j*_ by the *i*th market; and $\varepsilon_{ij}^{F}$ is an error term for each segment–product pair.

I adopt the SIMOPT model for cost calculation, which is proposed by Kwong et al. (2011). The cost function $C_{j}^{F}$ can be formulated as follows:

3 $$C_{j}^{F} = \mathop \sum \limits_{k = 1}^{K} \mathop \sum \limits_{l = 1}^{{L_{k} }} c_{kl}^{F} h_{jkl} , \quad \forall j$$

where $C_{j}^{F}$ is the cost of the *j*th product; $c_{kl}^{F}$ is the variable unit cost for the *l*th attribute level of the *k*th attribute.

And according to the MNL model, the choice probability $P_{ij}^{F}$ can be defined as Eq. (4), where *μ* is a scaling parameter. And *Q*_*i*_ is the size of the *i*th market segment.

4 $$P_{ij}^{F} = \frac{{{ \exp }\left( {\mu U_{ij}^{F} } \right)}}{{\mathop \sum \nolimits_{t = 1}^{T} { \exp }\left( {\mu U_{it} } \right)}}$$

One of the most important mechanism for this bi-level programming is the market share constraint, which is the interaction between leader and follower, as shown in Eq. (5).

5 $$\mathop \sum \limits_{j = 1}^{J} P_{ij}^{F} Q_{i} x_{j} \le Q_{i} - \mathop \sum \limits_{m = 1}^{M} P_{im}^{f} Q_{i} y_{m} ,\quad \forall i = 1,2, \ldots ,I$$

The upper-level constraints are mainly related to choice compatibility. For example, one attribute only can be chosen one corresponding level in one product. Other special constraints may be introduced as well. For example, a differentiation condition constraint limits each product in the product line should be different from others.

### Lower-level PPM optimization

The lower-level is optimization for the follower’s PPM problem. The decision variables of the lower-level are also two binary choice variables *y*_*m*_ (m = 1, 2, …, M) and *z*_*mns*_ (n = 1, 2, …, N; s = 1, 2, …, S_*n*_). Such that *y*_*m*_ = 1 means that product *r*_*m*_ is selected, and *y*_*m*_ = 0 not. While *z*_*mns*_ = 1 means that the *s*th attribute level of the *n*th attribute is contained in the *m*th product and *z*_*mns*_ = 0 not. Similarly to the leader, the follower’s objective is the construction of shared surplus, as shown in Eq. (6).

6 $$\mathop {\hbox{max} }\limits_{{y_{m} ,z_{mns} }} f = \mathop \sum \limits_{i = 1}^{I} \mathop \sum \limits_{m = 1}^{M} \frac{{U_{im}^{f} }}{{C_{m}^{f} }}P_{im}^{f} Q_{i} y_{m}$$

where $U_{im}^{f}$ is the utility of the *i*th segment for the *m*th product; $C_{m}^{f}$ is the cost of the *m*th product; $P_{im}^{f}$ is the choice probability; and *Q*_*i*_ is the size of the *i*th market segment. The constitution of these terms are as same as shown in “Upper-level PPM optimization” section.

The lower-level constraints are mainly about choice compatibility. For example, one attribute only can be chosen one corresponding level in one product. Other special constraints may be introduced as well. For example, a differentiation condition constraint limits each product in the product line should be different from others. The most important choice constraint is from the upper-level, it forms the mechanism of bi-level programming, defined as Eq. (5).

### Hierarchical joint optimization model

Compiling Eqs. (1)–(6), I can obtain the general form of hierarchical joint optimization for leader–follower PPM problem, as the following:

7a $$\mathop {\hbox{max} }\limits_{{x_{j} ,h_{jkl} }} F = \mathop \sum \limits_{i = 1}^{I} \mathop \sum \limits_{j = 1}^{J} \frac{{U_{ij}^{F} }}{{C_{j}^{F} }}P_{ij}^{F} Q_{i} x_{j}$$

7b $${\text{s}} . {\text{t}} .\quad U_{ij}^{F} = \mathop \sum \limits_{k = 1}^{K} \mathop \sum \limits_{l = 1}^{{L_{k} }} w_{jk}^{F} u_{ikl}^{F} h_{jkl} + \pi_{ij}^{F} + \varepsilon_{ij}^{F} , \quad \forall i,j$$

7c $$C_{j}^{F} = \mathop \sum \limits_{k = 1}^{K} \mathop \sum \limits_{l = 1}^{{L_{k} }} c_{kl}^{F} h_{jkl} , \quad \forall j$$

7d $$P_{ij}^{F} = \frac{{{ \exp }\left( {\mu U_{ij}^{F} } \right)}}{{\mathop \sum \nolimits_{t = 1}^{T} { \exp }\left( {\mu U_{it} } \right)}}$$

7e $$\mathop \sum \limits_{l = 1}^{{L_{k} }} h_{jkl} = 1,\quad \forall j,k$$

7f $$\mathop \sum \limits_{k = 1}^{K} \mathop \sum \limits_{l = 1}^{{L_{k} }} (h_{jkl} - h_{{j^{\prime}kl}} ) \ne 0,\quad \forall j,j^{\prime},j \ne j^{\prime}$$

7g $$\mathop \sum \limits_{j = 1}^{J} x_{j} \le J^{ + }$$

7h $$\mathop \sum \limits_{i = 1}^{I} \mathop \sum \limits_{j = 1}^{J} P_{ij}^{F} Q_{i} x_{j} \le \mathop \sum \limits_{i = 1}^{I} \left(Q_{i} - \mathop \sum \limits_{m = 1}^{M} P_{im}^{f} Q_{i} y_{m} \right)_{{}}$$

7i $$x_{j} \in \left( {0,1} \right)$$

7j $$h_{jkl} \in \left( {0,1} \right)$$

7k $$\mathop {\hbox{max} }\limits_{{y_{m} ,z_{mns} }} f = \mathop \sum \limits_{i = 1}^{I} \mathop \sum \limits_{m = 1}^{M} \frac{{U_{im}^{f} }}{{C_{m}^{f} }}P_{im}^{f} Q_{i} y_{m}$$

7l $${\text{s}} . {\text{t}} .\quad U_{im}^{f} = \mathop \sum \limits_{n = 1}^{N} \mathop \sum \limits_{s = 1}^{{S_{n} }} w_{mn}^{f} u_{mns}^{f} z_{mns} + \pi_{im}^{f} + \varepsilon_{im}^{f} , \quad \forall i,m$$

7m $$C_{m}^{f} = \mathop \sum \limits_{n = 1}^{N} \mathop \sum \limits_{s = 1}^{{S_{n} }} c_{ns}^{f} z_{mns} , \quad \forall m$$

7n $$P_{im}^{f} = \frac{{{ \exp }\left( {\mu U_{im}^{f} } \right)}}{{\mathop \sum \nolimits_{t = 1}^{T} { \exp }\left( {\mu U_{it} } \right)}}$$

7o $$\mathop \sum \limits_{s = 1}^{{S_{n} }} z_{mns} = 1,\quad \forall m,n$$

7p $$\mathop \sum \limits_{n = 1}^{N} \mathop \sum \limits_{s = 1}^{{S_{n} }} (z_{mns} - z_{{m^{\prime}ns}} ) \ne 0,\quad \forall m,m^{\prime } ,m \ne m^{\prime }$$

7q $$\mathop \sum \limits_{m = 1}^{M} y_{m} \le M^{ + }$$

7r $$\mathop \sum \limits_{i = 1}^{I} \mathop \sum \limits_{m = 1}^{M} P_{im}^{f} Q_{i} y_{m} \le \mathop \sum \limits_{i = 1}^{I} (Q_{i} - \mathop \sum \limits_{j = 1}^{J} P_{ij}^{F} Q_{i} x_{j} )_{{}}$$

7s $$y_{m} \in \left( {0,1} \right)$$

7t $$z_{mns} \in \left( {0,1} \right)$$

The upper-level controls the lower-level through *X* and *H*, indicating a priority for determining PPM. The lower-level entails a nonlinear parametric programming problem, in which (*X, H*) are parametric variables, suggesting that parametric optimization must be compatible with its own PPM problem. The lower-level returns an optimum function $P_{im}^{f} \left( {z_{mns} } \right)$ as the feedback to the upper-level. Optimal solution (X*, H*, Y*, Z*) describes the design schemes for leader and follower, where X* and H* indicate the leader’s solutions and Y*, Z* are follower’s.

## Model solution

Solution of bi-level programming tends to be much more complex than single-level optimization problems (Christiansen et al. 2001; Bard 1998). Bi-level programming solutions are generally categorized as direct and indirect methods: solving the bi-level programming directly or converting the bi-level programming to a single-level programming. Direct methods comply with the bi-level decision mechanism, starting with the upper-level and find one solution first. Then the lower-level uses the corresponding upper-level variables as parameters to solve the lower-level problem.

Model (7) is a nonlinear programming with 0–1 variables, which is proved to be a NP-hard problem. And essentially this hierarchical product portfolio management problems entail combinational optimization that can be solved by genetic algorithms (Oliveto et al. 2007). Comparing with traditional calculus-based or approximation-based optimization techniques, GA is excellent in solving combinatorial optimization problems (Kreng and Lee 2004). Due to the distinctive characteristics of the upper- and lower-level optimization problems, I propose two separate GAs for the respective upper and lower levels, while the upper- and lower-level GAs are nested through a constraint handling mechanism embedded through GA encoding and GA operators. Specially, in the bi-level nested GA operations, I address two fitness functions which are consistent with their objective functions of upper- and lower-level, respectively. This kind of fitness function can identify the quality of different potential solutions.

### GA encoding

**Fig. 2:**
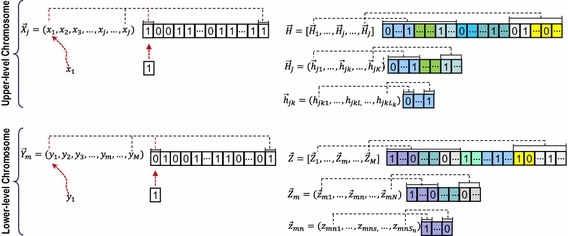
Bi-level nested GA encoding for this optimization

The very basis of GA implementation is about representation of the problem to be solved with a finite-length string called a chromosome. Consistent with the bi-level decision-making of leader–follower PPM problem, two kinds of chromosomes are composed in this bi-level nested GA: the upper-level chromosomes (ULCs) and the lower-level chromosome (LLCs), While ULCs and LLCs represent solution *X*, *H* and *Y, Z*, respectively. As for the ULCs, the length of chromosome *X* is the total number of products *J*, and each gene in this chromosome represents a product; and the length of chromosome *H* is J*K*L_*k*_, and each gene in this chromosome means an attribute level. Similarly, the length of chromosome *Y* is *M* and each gene means the choice of product; and the length of chromosome *Z* is M*N*S_*n*_, and each gene represents an attribute level in lower-level. Figure 2 shows the bi-level nested GA encoding.

### GA crossover and mutation

**Fig. 3:**
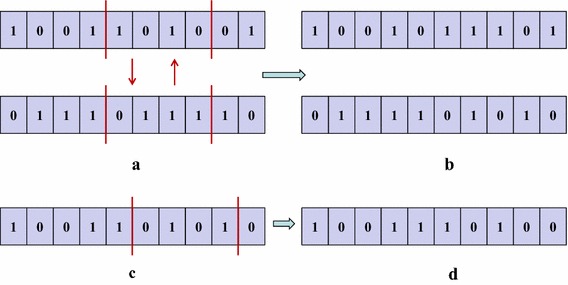
Crossover and mutation for the ULC. **a** Choice of crossover points, **b** children by crossover, **c** choice of mutation points, **d** child by mutation

To obtain feasible solutions, chromosomes crossover and mutation should be occurred on the basis of satisfying certain design constraints. Crossover in GA operations means that optional two parent chromosomes are randomly chosen to exchange the segments of their genes with a probability. Figure 3a illustrates the crossover mechanism for the ULC *X*. Once a pair of ULC *X* chromosomes is chosen, two crossover points are generated randomly for each of them, and the exchange range is specified in the meantime. As shown in Fig. 3b, the children chromosomes are acquired by exchanging the parts of the selected chromosomes.

Mutation sockets in each offspring individually after crossover. It randomly selects a gene with a smaller probability and alters the selection or de-selection of corresponding module instances in a chromosome. Figure 3c shows that how the mutation points are generated randomly. Finally the selected chromosome segment is changed by a random number of modules, as shown in Fig. 3d.

### GA nested solution flow

**Fig. 4:**
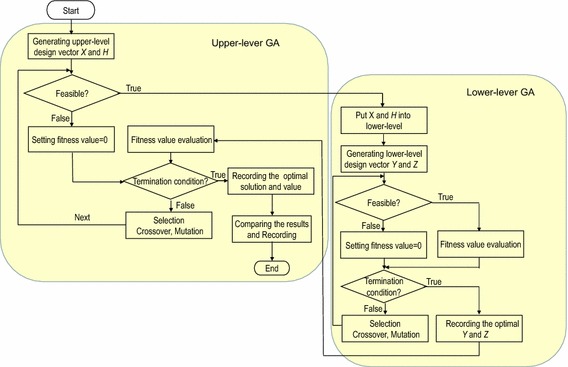
The solution flow of the bi-level nested GA

Solving nonlinear bi-level programming of PPM joint optimization relies on principles of evolutionary computation, videlicet the nested genetic evolutions between upper- and lower-level optimization. The bi-level GA with the aforementioned encoding schemas and operators is carried out in a nested way, as shown in Fig. 4. A step-by-step procedure for the bi-level nested algorithm is described as follows:

- *Step 1* A population of size *N* is generated with the required number of upper-level variables, randomly. Constraint checking is carried out to ensure that the initial population satisfies all design rules specified in the model. Subsequently, the lower-level GA procedure is launched to search the corresponding optimal values of the lower-level variables. If constraint checking fails, set the fitness values to be zero then turn to the next step.
- *Step 2* Judge whether the population reaches the maximum number of generation. If the GA runs for the maximal number of generations, record the optimal value and then go to the next step. Otherwise, GA operations of selection, crossover and mutation are carried out; and then go back to Step 1.
- *Step 3* Take the upper-level solution *X* and *H* into the lower-level GA. Verify feasibility of the lower-level population. The fitness is evaluated when the feasibility is satisfied. Otherwise, the fitness value is set to zero.
- *Step 4* Estimate whether lower-level population achieves the maximum number of generations. If it reaches the upper bound, the optimal variables and optimal value are recorded. Otherwise, procedures of selection, crossover and mutation are invoked and step 3 is repeated till the maximal number of generations is achieved.
- *Step 5* Echo the lower-level solution *Y* and *Z* back to the upper-level GA. The upper-level GA is re-calculated and the fitness values are re-evaluated. Repeat Step 4 until the lower-level population reaches the maximum number of generations.
- *Step 6* Check the termination condition of the lower-level GA. If a feasible number is determined for the entire modules, the upper- and lower-level optimal values are recorded and then the iterative procedure ends. Otherwise, proceed to Step 1 and repeat the process.

## Case study

A case study of PPM optimization problem for the notebook computer products is reported to demonstrate the potential of the hierarchical joint optimization model. The product structure of notebook computer mainly consists of display, hard disk, graphics card, processer, battery and memory, etc.

### Upper-level

Based on the service handbook and domain expertise, all the attributes and feasible attribute levels and their corresponding costs of notebook computer for leader are listed in Table 1.

**Table 1:** List of attributes and their levels of upper-level

No.	Attribute	Code	Attribute levels	Cost
A1	Display	A11	13.3″ HD	248
		A12	14.0″ HD	330
		A13	15.6″ HD	450
A2	Hard disk	A21	250 GB 5400 rpm hard drive	255
		…	…	…
		A25	128 GB solid state drive	580
A3	Graphics card	A31	Intel HD graphics	659
		…	…	…
		A35	NVIDIA GTX860 M 4 GB	2290
A4	Main board	A41	B85	890
		…	…	…
		A43	X99	2580
A5	Processer	A51	Intel core i5, 2 cores	1460
		…	…	…
		A55	Intel core i7, 6 cores	2855
A6	Battery	A61	6-Cell lithium-ion	440
		A62	9-Cell lithium-ion	635
A7	Memory	A71	4G DDR3	248
		A72	8G DDR3	380
A8	Case	A81	Metal	170
		A82	ABS	96

To specify utility function $U_{ij}^{F}$ in upper-level, $w_{jk}^{F} , u_{ikl}^{F}$ and $\pi_{ij}^{F}$ should be confirmed first. $w_{jk}^{F}$ can be determined up to market survey; $u_{ikl}^{F}$ can be obtained by conjoint analysis; and $\pi_{ij}^{F}$ is formed from $u_{ikl}^{F}$. Conjoint analysis is a market survey method for product design. According to conjoint analysis method, the first step to calculate $u_{ikl}^{F}$ is to given a set of product which contains the total number of *J* possible combinations. Then 20 orthogonal setup of product profiles can be generated using the Taguchi Orthogonal Array Selector in SPSS software, as shown in Table 2. While inviting 60 consumers to do a fractional factorial experiment with the 20 products. The result of conjoint analysis is shown in Table 3. For illustrative simplicity without losing the context, I consider one leading market for this leader–follower PPM problem (*I* = 1), also assume that *Q*_*i*_ = 10,000, and J^+^ = 4.

**Table 2:** Orthogonal setup of product profiles for conjoint analysis of upper-level

#	Display	Hard disk	Graphics card	Main board	Processer	Battery	Memory	Case
1	13.3″ HD	500 GB	Intel HD graphics	B85	Intel core i5, 2 cores	2–3 h	4G DDR3	ABS
2	14.0″ HD	500 GB	NVIDIA GTX540 M 2 GB	B85	Intel core i5, 2 cores	2–3 h	4G DDR3	ABS
…	…	…	…	…	…	…	…	…
20	15.6″ HD	128 G Solid	NVIDIA GTX860 M 4 GB	X99	Intel core i7, 6 cores	Above 5 h	8G DDR3	Metal

**Table 3:** Part-worth utilities of upper-level

Attribute	Code	Attribute levels	$u_{ikl}^{F}$
Display	A11	13.3″ HD	1.69
	A12	14.0″ HD	1.87
	A13	15.6″ HD	2.25
Hard disk	A21	250 GB 5400 rpm hard drive	1.45
	…	…	…
	A25	128 GB solid state drive	1.76
Graphics card	A31	Intel HD graphics	2.34
	…	…	…
	A35	NVIDIA GTX860 M 4 GB	3.19
Main board	A41	B85	1.92
	…	…	…
	A43	X99	3.40
Processer	A51	Intel core i5, 2 cores	2.58
	…	…	…
	A55	Intel core i7, 6 cores	4.23
Battery	A61	6-Cell lithium-ion	−0.82
	A62	9-Cell lithium-ion	1.58
Memory	A71	4G DDR3	2.23
	A72	8G DDR3	2.47
Case	A81	Metal	−0.57
	A82	ABS	1.61

Meanwhile, other compatibility problems, such as, the consistent matching of processer and memory, display and battery should be formulated as constraint equations, as the following:

8 $$h_{j55} \ne h_{j72}$$

9 $$h_{j13} = h_{j62}$$

### Lower-level

The lower-level PPM optimization problem is as same as the upper-level, but the parameters of products are different for their diverse production capacity and market positioning. The attributes, attribute levels and the corresponding costs of products of the follower are shown in Table 4.

**Table 4:** List of attributes and their levels of lower-level

No.	Attribute	Code	Attribute levels	Cost
B1	Display	B11	13.3″ HD	276
		B12	14.0″ HD	360
		B13	15.6″ HD	510
B2	Hard disk	B21	250 GB 5400 rpm hard drive	294
		…	…	…
		B24	2T 7200 rpm hard drive	488
B3	Graphics card	B31	Intel HD graphics	718
		…	…	…
		B34	NVIDIA GTX860 M 4 GB	2440
B4	Main board	B41	B85	920
		…	…	…
		B43	X99	2710
B5	Processer	B51	Intel core i5, 2 cores	1730
		…	…	…
		B54	Intel core i7, 6 cores	3005
B6	Battery	B61	6-Cell lithium-ion	475
		B62	9-Cell lithium-ion	680
B7	Memory	B71	4G DDR3	266
		B72	8G DDR3	410
B8	Case	B81	Metal	191
		B82	ABS	104

And with the same conjoint analysis method in “Upper-level” section, I can obtain a set of 20 orthogonal setup of product profiles and the part-worth utilities, are separately shown as Tables 5 and 6.

**Table 5:** Orthogonal setup of product profiles for conjoint analysis of lower-level

#	Display	Hard disk	Graphics card	Main board	Processer	Battery	Memory	Case
1	13.3″ HD	250 GB	Intel HD graphics	B85	Intel core i5, 2 cores	2–3 h	4G DDR3	ABS
2	14.0″ HD	500 GB	NVIDIA GTX540 M 2 GB	B85	Intel core i5, 4 cores	2–3 h	4G DDR3	ABS
…	…	…	…	…	…	…	…	…
20	15.6″ HD	2T	NVIDIA GTX860 M 4 GB	X99	Intel core i7, 4 cores	Above 5 h	8G DDR3	Metal

**Table 6:** Part-worth utilities of lower-level

Attribute	Code	Attribute levels	$u_{ikl}^{F}$
Display	B11	13.3″ HD	1.54
	B12	14.0″ HD	1.79
	B13	15.6″ HD	2.33
Hard disk	B21	250 GB 5400 rpm hard drive	1.62
	…	…	…
	B24	2T 7200 rpm hard drive	1.81
Graphics card	B31	Intel HD graphics	2.22
	…	…	…
	B34	NVIDIA GTX860 M 4 GB	2.99
Main board	B41	B85	1.47
	…	…	…
	B43	X99	2.69
Processer	B51	Intel core i5, 2 cores	2.33
	…	…	…
	B54	Intel core i7, 6 cores	3.83
Battery	B61	6-Cell lithium-ion	−0.82
	B62	9-Cell lithium-ion	1.58
Memory	B71	4G DDR3	2.85
	B72	8G DDR3	2.97
Case	B81	Metal	−0.89
	B82	ABS	1.22

And I also assume that the number of products M^+^ = 4. The very important constraint in the lower-level is from the upper-level. The constraint $\mathop \sum \nolimits_{m = 1}^{M} P_{im}^{f} Q_{i} y_{m} \le Q_{i} - \mathop \sum \nolimits_{j = 1}^{J} P_{ij}^{F} Q_{i} x_{j} , \,\forall i = 1,2, \ldots ,I$ means that the market size for the follower is not the whole size of market segment but the rest part from the leader’s market share.

Similarly, according to the practical situation of product technical design, other technical constraints, such as, attribute level B13 is not compatible with B61, can be given by Eqs. (10)–(12).

10 $$z_{m13} \ne z_{m61}$$

11 $$z_{m24} \ne z_{m41}$$

12 $$z_{m33} \ne z_{m42}$$

### Solution and results

I adopt a bi-level nested GA to solve this optimization model which is a nonlinear programming with 0–1 variables. For each feasible chromosome of upper-level population, the corresponding lower-level optimal value is sent back to the upper-level, then the upper-level fitness value is calculated, as shown in Fig. 4. According to Jiao (2007, 2013), for the more complex combination of attribute model, the fitness population size is 100 for GA.

**Fig. 5:**
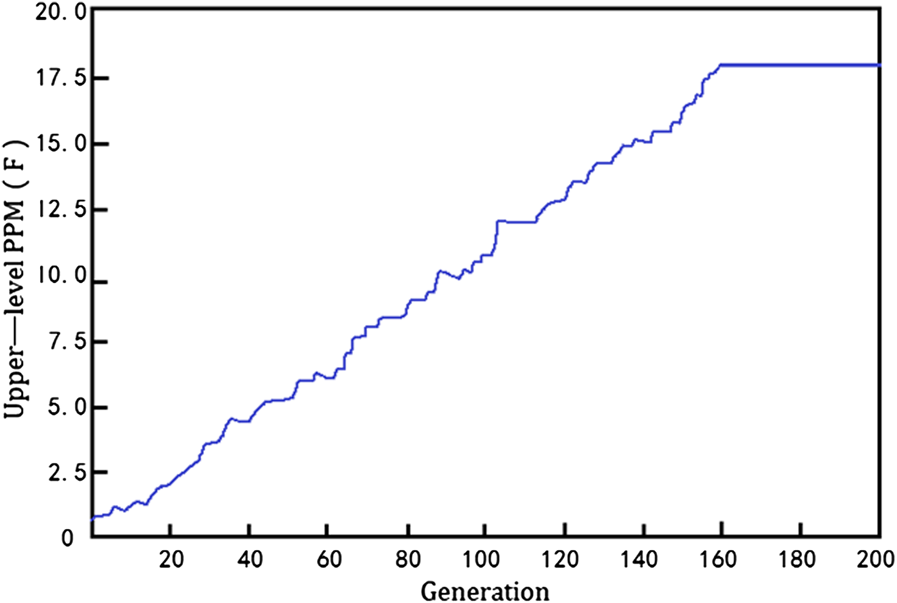
Bi-level nested GA optimization process

For a medium-scale combinatorial configuration problem like this notebook computer product portfolio, I empirically set the population size at 100 for both the upper- and lower-level, crossover probability is 0.6, mutation probability is 0.005, and the generation is 200. Figure 5 shows the GA processes with the upper-level, after around 160 iterations the calculation verges to convergence. Similar outputs of the lower-level GA for PPM can be obtained as well.

**Fig. 6:**
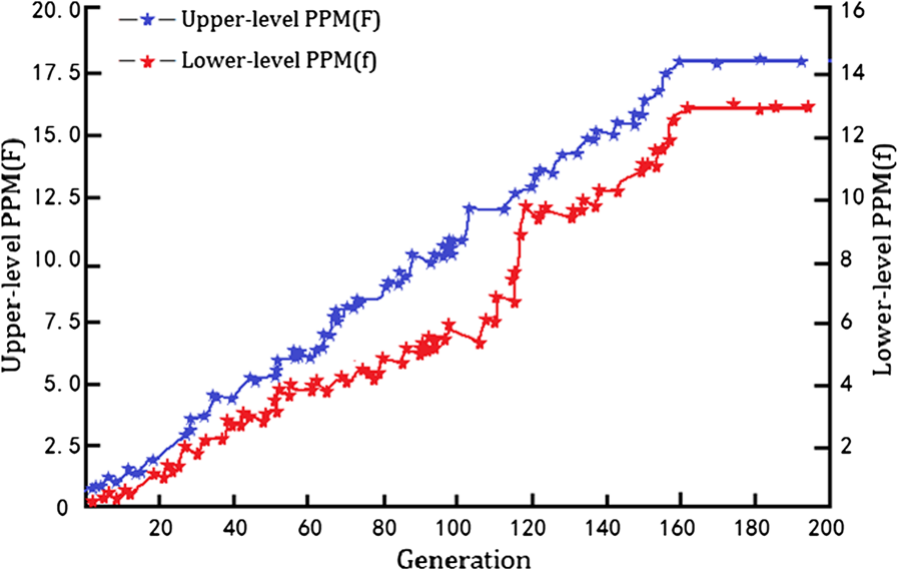
Nested convergence of the upper- and lower-level

Figure 6 shows how the upper- and lower-level GAs are nested during their convergence processes, indicating the leader’s and follower’s PPM problems are competing during the optimal design of notebook computers. Finally after around 160 generations, both the upper- and lower-level fitness values reach their optima, suggesting that the leader’s and follower’s PPM problems arrive at equilibrium solutions of the Stackelberg game. The optimal product portfolios for leader and follower are shown in Tables 7 and 8.

**Table 7:** The leader’s optimal product portfolio

Attribute	Product variant			
	Product 1	Product 2	Product 3	Product 4
Display	13.3″ HD	14.0″ HD	15.6″ HD	15.6″ HD
Hard disk	1T 5400 rpm hard drive	1T 5400 rpm hard drive	2T 7200 rpm hard drive	128 GB solid state drive
Graphics card	Intel HD graphics	Intel HD graphics	NVIDIA GTX540 M 2 GB	NVIDIA GTX860 M 4 GB
Main board	B85	Z97	X99	X99
Processer	Intel core i5, 2 cores	Intel core i5, 2 cores	Intel core i7, 4 cores	Intel core i7, 6 cores
Battery	6-Cell lithium-ion	9-Cell lithium-ion	9-Cell lithium-ion	9-Cell lithium-ion
Memory	4G DDR3	4G DDR3	8G DDR3	8G DDR3
Case	ABS	Metal	Metal	Metal

**Table 8:** The follower’s optimal product portfolio

Attribute	Product variant			
	Product 1	Product 2	Product 3	Product 4
Display	13.3″ HD	13.3″ HD	14.0″ HD	14.0″ HD
Hard disk	500 GB 5400 rpm hard drive	1T 5400 rpm hard drive	2T 7200 rpm hard drive	2T 7200 rpm hard drive
Graphics card	Intel HD graphics	Intel HD graphics	NVIDIA GTX540 M 2 GB	NVIDIA GTX860 M 4 GB
Main board	B85	B85	Z97	X99
Processer	Intel core i5, 2 cores	Intel core i7, 4 cores	Intel Core i7, 4 cores	Intel core i7, 6 cores
Battery	6-Cell lithium-ion	6-Cell lithium-ion	9-Cell lithium-ion	9-Cell lithium-ion
Memory	4G DDR3	4G DDR3	8G DDR3	8G DDR3
Case	ABS	ABS	Metal	Metal

### Performance analysis

#### The optimal design

Examining the results of leader–follower PPM joint optimization in Tables 7 and 8 indicate that the optimal design of notebook computer product portfolios are logical and integrate as market-oriented products. The joint optimization results in Table 9 indicate a reasonable leverage of leader and follower. I can also figure out that the nonlinear bi-level programming method can benefit both leader and follower well due to its decision-making mechanism by considering the market compititive factors and engineering parameters in PPM. Meanwhile this method can also gain a superior globle design scheme and it is better than non-joint one because this bi-level joint optimization correctly discribes the gaming relationship betweeen leader and follower in a market place.

**Table 9:** Optimal values of the upper- and lower-level

Comparison	Evaluation criteria		
	Utility	Cost (RMB)	Objective function
Upper-level	68.84	20,781	17.73
Lower-level	60.59	21,524	12.89

#### The algorithms

To demonstrate the advantages of the nested GA, computational experiments are set up to compare its performance with other relevant algorithms. Related research has shown that genetic algorithm, simulated annealing algorithm and ATC methods perform well in product line design and product portfolio management problems (Balakrishnan et al. 2004; Belloni et al. 2008; Michalek et al. 2011). For the ATC method can only handles continuous variables, I just compare the former two methods for the scenario of *J* = *4*, *M* = *4*. And all the computations were conducted in Matlab on a Core i5 personal notebook computer.

Table 10 provides the results of comparison. I can figure out that no matter in terms of CPU time or Optimization objective, GA is much more efficient than SA method. These results suggest that, GA is more suitable for solving the PPM bi-level programming in this case.

**Table 10:** Algorithms comparisons for bi-level programming

Evaluation criteria	Comparison			
	GA		SA	
CPU time (h)	1.8		77.4	
Optimization objective	Upper-level	Lower-level	Upper-level	Lower-level
	17.73	12.89	16.99	12.01

## Conclusions

PPM is different from the traditional single product or product line design problem, because it must handle a group of products with simultaneously optimizing the attributes of each product variant. The proposed nonlinear bi-level programming model which is applied to maximize the shared surplus of both leader and follower emphasizes the competitive relationships and tradeoffs between the leader and follower in leveraging their PPM problems. This method provides a new perspective to optimize PPM problem with different decision makers from both theory and practice. The bi-level nested genetic algorithm coincides with the bi-level decision structure of joint optimization. Encoding of attribute and product selection through a binary decision variable of each gene contributes to efficient solution of a class of non-linear combinatorial optimization programs. Results and analysis of the notebook computer case study demonstrate that the nonlinear bi-level optimization model is more reasonable and excellent in supporting leader–follower optimization for PPM problem and the proposed bi-level nested GA method is efficient for solving this challenging programming. Moreover, the introduced bi-level optimization framework and method can also be applied to other areas, such as, engineering design problem with multiple conflictive objects, supply chain coordination and optimization problem, and other optimization problems with game-theory relations.

Future research could focus on extending the bi-level programming model to multiple perspectives in PPM problem. With the considerations of the actual background in a competitive market, it could be a one-leader multi-follower bi-level programming for PPM problem. Solving such multi-follower model could be a very challenging issue that calls for both theoretical observation and efficient solution algorithms.
